# Bilateral Endogenous Endophthalmitis With Klebsiella pneumoniae Bacteremia Secondary to Hepatic Abscess

**DOI:** 10.7759/cureus.66287

**Published:** 2024-08-06

**Authors:** Amith Rao, Megan K Taylor, Tom Marco, Zachary Chun

**Affiliations:** 1 Department of Internal Medicine, University of Arizona College of Medicine - Tucson, Tucson, USA

**Keywords:** ophthalmology ocular pathology, klebsiella pneumoniae, ophthalmology, bacteremia, klebsiella, klebsiella liver abscess, endogenous klebsiella endophthalmitis, infectious disease pathology

## Abstract

*Klebsiella* endophthalmitis is a rare cause of endogenous endophthalmitis, with very few cases documented in the US. We present a male patient in his 60s with a history of latent tuberculosis who presented to the hospital with complaints of acute bilateral vision loss that began three days prior to admission. The workup revealed *Klebsiella pneumoniae* bacteremia, a large hepatic abscess, severe orbital swelling, and acute angle-closure glaucoma. The patient received intravitreal antibiotics, intravenous antibiotics, a hepatic drain, intraocular pressure-lowering medications, and steroids. Bacteremia was cleared with antibiotics and source control; however, vision loss did not improve. This case emphasizes the acuity and severity of *Klebsiella* endogenous endophthalmitis and outlines the need for immediate intervention with the onset of symptoms to prevent irreversible vision loss.

## Introduction

*Klebsiella pneumoniae* (*K. pneumoniae*), a member of the *Enterobacteriaceae* family, is characterized as a gram-negative bacterium. Its virulence lies in its capsule, pili, exopolysaccharides, lipopolysaccharides, and adhesion, all of which enable evasion from the host’s innate immune system [1]. Humans are the primary hosts of this bacterium, and the organism typically resides in the nasopharynx and gastrointestinal tract. Due to its virulence factors, it can cause multiple life-threatening infections, including pneumonia, abscesses, urinary tract infections, and much less commonly endogenous endophthalmitis (EE) [2]. Systemic disease has been reported in up to 16% of *K. pneumoniae *liver abscess cases [2].

Over the last several decades, liver abscesses caused by hypervirulent *Klebsiella pneumoniae* (HvKP) strains have become increasingly prevalent in Asia, estimated to consist of 80% of all pyogenic liver abscesses in Taiwan and Korea [3]. In the United States (US), studies report an incidence ranging from 7% to 27% [4]. HvKP are more likely to cause disseminated infections and are more frequently associated with liver abscess, endophthalmitis, meningitis, and necrotizing fasciitis. When grown on an agar plate, these colonies have a hypermucoviscous appearance and are characterized by a positive string test, which is demonstrated by a viscous string measuring more than 5 millimeters (mm) in length when stretching bacterial colonies using an inoculation loop.

EE involves intraocular inflammation caused by metastatic dissemination from systemic infections. It is thought to occur through septic emboli that enter the posterior segment vasculature of the eye. Due to the significant inflammation within the eye, the most common symptoms are vision loss, conjunctival injection, and the presence of floaters. The incidence of EE in patients with *Klebsiella* liver abscesses has been largely unknown, as many studies have analyzed rates of endophthalmitis in the setting of pyogenic liver abscesses without distinguishing between *Klebsiella* and other bacteria. A meta-analysis from 2020 retrospectively analyzed the incidence of EE in 12,000 patients from East Asia with *K. pneumoniae* pyogenic liver abscess and found a statistically significant rate of 4.5% of these patients who later developed EE [5]. This report illustrates a case involving a male in his 60s who presented for bilateral visual loss and was found to have hypervirulent *Klebsiella pneumoniae* bacteremia with associated liver abscess, EE, and septic emboli to the brain.

## Case presentation

A male patient in his 60s with a history of latent tuberculosis presented to the emergency room with complaints of bilateral vision loss and generalized malaise. The patient reported feeling at his usual state of health until five days prior to the presentation. He complained of a productive cough with yellow sputum, shortness of breath, chest pain with inspiration, lower abdominal pain that radiated to his back, nausea, and myalgias. Three days prior to the presentation, he fell asleep in his car and woke up with acute-onset bilateral vision loss. He reported one sick contact - his mother recently tested positive for influenza. Though he grew up in Mexico, he had not returned over the last 40 years and denied any recent travel outside of the US. The patient works as a janitor and denied any history of tobacco use, alcohol use, or intravenous (IV) drug use.

**Figure 1:**
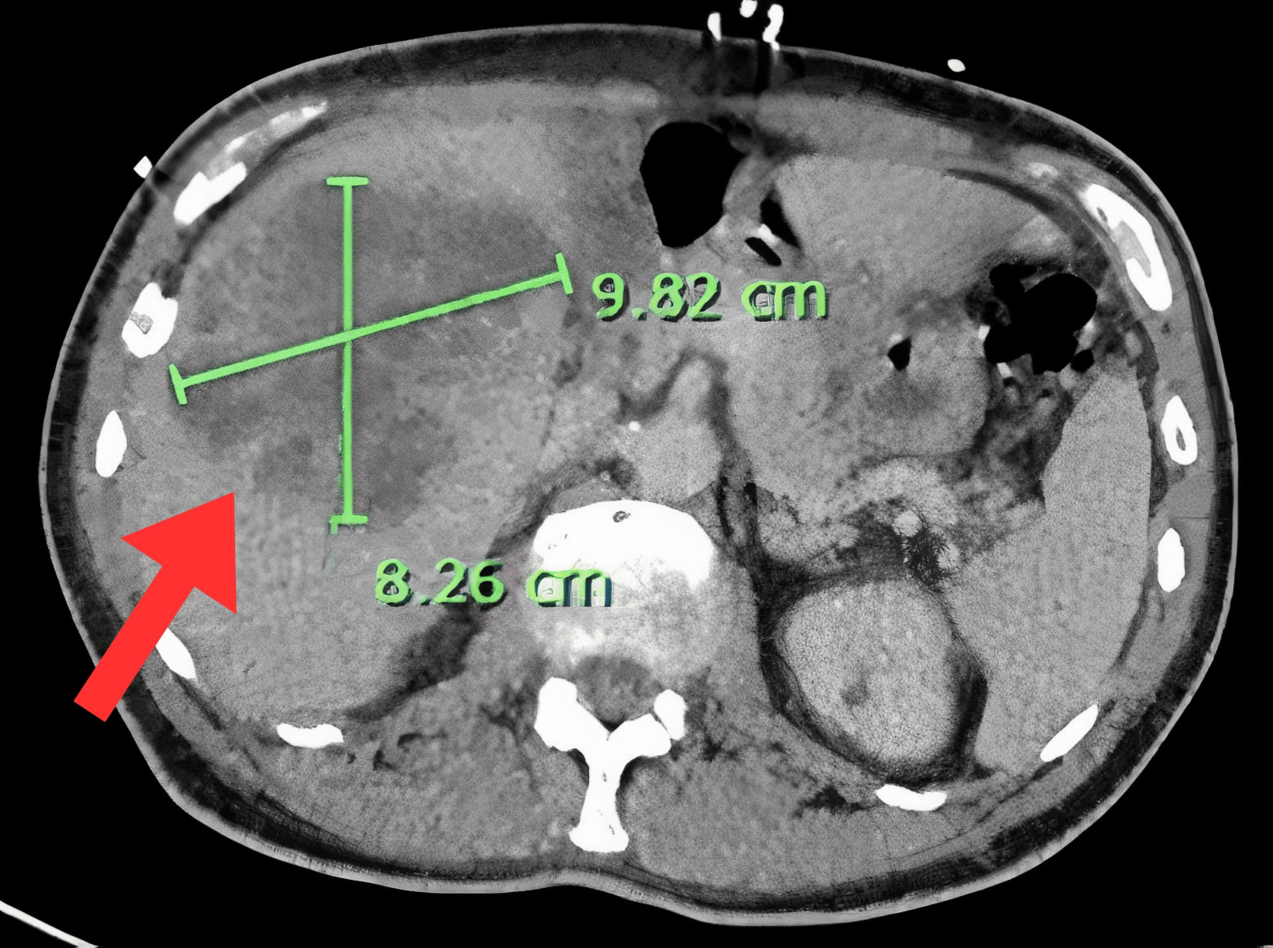
CT of the abdomen/pelvis with contrast done on admission showed a large multiloculated heterogeneous mass (red arrow) within the liver measuring 9.82 x 8.26 cm.

In the emergency department, the patient was found to have tachypnea and hypoxia requiring 2 liters of supplemental oxygen. Lab investigations were notable for leukocytosis, lactic acidosis, and mildly elevated liver enzymes. Computed tomography (CT) of the head was negative. CT angiography (CTA) of the thorax showed bronchiectasis and consolidations concerning pneumonia. CT of the abdomen/pelvis showed a 9.82 x 8.26 cm multiloculated heterogeneous mass within the liver, concerning for an abscess (Figure 1). Ophthalmology evaluated and noted significant vitreous opacity and debris with no view to the fundus, diagnosing him with severe endogenous endophthalmitis of both eyes. The patient received one-time intravitreal antibiotics (vancomycin 1 milligram (mg)/0.1 milliliter (mL) and ceftazidime 2 mg/0.1 mL) and intravitreal dexamethasone 400 micrograms (mcg)/0.1 mL injections. Vitreous cultures were obtained. Bilateral eye drops were also administered, including moxifloxacin four times daily for seven days, prednisolone acetate twice daily for seven days, and atropine daily for three days.

Subsequently, the patient developed septic shock requiring intubation and admission to the medical intensive care unit (ICU). A percutaneous hepatic drain was placed by the interventional radiology team one day after admission, and at that time, the patient was started on empiric IV meropenem 500 mg every six hours, vancomycin 750 mg every 12 hours, and azithromycin 500 mg daily. Two days later, blood cultures and liver abscess fluid cultures grew Klebsiella pneumoniae susceptible to ceftriaxone. Infectious disease was consulted. At that time, systemic antibiotics were narrowed to ceftriaxone 2 grams (g) IV daily, and meropenem, vancomycin, and azithromycin were discontinued. While sputum cultures grew methicillin-sensitive Staphylococcus aureus, this was presumed to be a contaminant, and the patient's pneumonia later resolved with this regimen. The patient was weaned off pressors and sedation and then extubated five days into the hospital course.

**Figure 2:**
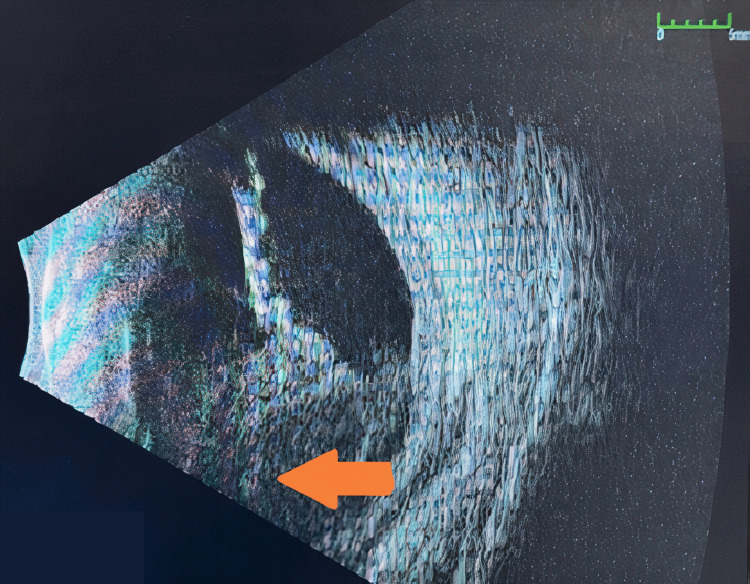
Right eye ocular ultrasound showing hyperechoic stranding in the vitreous cavity (orange arrow) along with serous choroidal detachment, suggestive of endogenous endophthalmitis.

**Figure 3:**
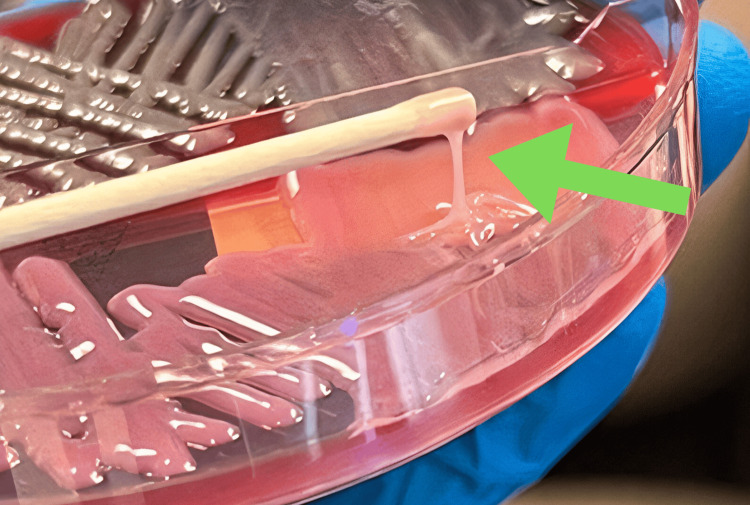
Positive string test (green arrow) of the patient indicating likely hypervirulent strain of Klebsiella pneumoniae.

After recovery from septic shock and weaning of sedation, the patient reported his vision was “white” with associated bilateral ocular pain. The ophthalmologic exam appeared significantly worse with new venous congestion and chemosis. A B-scan was performed with evidence of serous bilateral choroidal detachments (Figure 2). CT of the brain/orbit showed bilateral scleral thickening and choroidal enhancement, mild post-septal fat stranding with proptosis, and questionable left optic nerve enhancement. Given these ocular findings, ceftriaxone 2 g was increased to every 12 hours for better CNS penetration for a 10-day course. The *Klebsiella pneumoniae* growing in the blood cultures was determined to be HvKP based on a positive string test performed by the infectious diseases team (Figure 3). An HIV test was negative.

The following day, the patient developed severely elevated intraocular pressures (IOPs) due to acute angle closure likely secondary to the choroidal detachments and subsequent anterior rotation of the lens-ciliary body diaphragm. IOP resolved with multiple rounds of aqueous suppressant eye drops and IV acetazolamide. Magnetic resonance imaging (MRI) of the brain/orbit showed evidence of septic emboli in the brain, presumed to be from a hepatic abscess. Two separate transthoracic echocardiograms were negative for endocarditis and intracardiac shunt. Systemic oral corticosteroids (prednisone 60 mg daily for seven days) were started and subsequent serial IOPs and a repeat B scan showed clinical improvement. The etiology of the choroidal detachments was presumed to be secondary to endogenous endophthalmitis. Vitreous cultures ultimately showed no growth.

Ceftriaxone IV 2 g twice daily was continued for one month total and then switched to ceftriaxone IV 2 g daily for an additional one month. Prednisone was tapered as follows: 40 mg for one week, then 20 mg for one week, then 10 mg for one week, and then 5 mg for one week. Repeat MRI of the orbit/brain approximately one month after the initial presentation showed the septic emboli had decreased from 5 mm to 4 mm and showed interval improvement of previous foci of intracranial enhancement. Repeat CT of the abdomen/pelvis around the same time showed an unchanged hepatic abscess. The hepatic drain was repositioned with subsequent improvement of the abscess on repeat scans, and eventually underwent removal of the drain. By the end of the hospitalization, the patient reported mild improvement in bilateral vision, reporting the ability to see a little more color and outlines of shapes but still functionally blind. The patient was discharged home with close ophthalmology and infectious disease outpatient follow-up. A peripherally inserted central catheter (PICC) line was placed so that the patient could continue IV ceftriaxone 2 g daily as above. He continued prednisone taper at home and was prescribed topical dorzolamide four times daily.

## Discussion

This report documents the case of a male in his 60s with a history of latent tuberculosis who presented for bilateral visual loss and was found to have HvKP bacteremia with associated liver abscess, EE, and septic emboli to the brain. Despite rapid management, the patient remained with relatively unchanged vision loss by the end of his hospitalization. Studies have shown that vision loss resulting from EE with late presentation is irreversible [6]. This case emphasizes the importance of avoiding any delays in treatment while awaiting culture results.

The current guidelines for *Klebsiella* bacterial EE include intravitreal antibiotics plus systemic antibiotics, with the option of vitrectomy based on the clinical condition. Intravitreal antibiotic therapy should include a combination of 3rd-generation cephalosporins and/or vancomycin and/or aminoglycoside [7]. A second injection should be administered if vision worsens 24 to 48 hours after the first dose [8]. A third injection may be considered if vision still does not improve after this same time interval. This is because antibiotics are rapidly metabolized in the vitreous, with the concentration being observed to last 24 to 48 hours. Our patient reported unchanged visual defects and therefore received only one round of injection.

Surgical management is often preferred as it is associated with improved visual outcomes and permits direct debridement of infected vitreous tissue. Connell et al. [8] showed that patients with *Klebsiella* endophthalmitis experience better outcomes with vitrectomy in a prospective case series. They reported an evisceration and enucleation rate of 0% in patients receiving vitrectomy and intravitreal antibiotics, in comparison to a rate of 50% when receiving intravitreal antibiotics only. This poses a challenge as patients with bacteremia are also seen as unfit for surgery. Throughout his hospitalization, this patient experienced hemodynamic instability and coagulopathy (thrombocytopenia), was intubated for hypoxic respiratory failure due to pneumonia, and therefore surgery was not pursued due to the high surgical risk. Furthermore, some advocate against vitrectomy due to the potential risk of retinal damage as well as reduced exposure of the infected retina to the intravitreal antibiotic since the vitreous body is no longer present [7]. Delayed vitrectomy likewise has not demonstrated clear benefits as EE often involves rapid deterioration; one retrospective analysis of streptococcal EE by Yospaiboon et al. revealed that early vitrectomy within three days of diagnosis was the only positive predictive factor for improved visual outcomes [9]. Ultimately, clinicians must weigh the risks versus benefits of surgery on an individual patient basis.

EE generally presents with a vague array of visual symptoms, such as pain, irritation, visual spots, and/or vision loss, which can make it challenging to diagnose and may contribute to delays in seeking care in the cases of less severe symptoms. Ultimately, this case proposes a question of whether ocular screening should be considered in patients with sepsis secondary to *Klebsiella pneumoniae*, with or without visual symptoms. A study by Chung et al. argues that eye screening should be incorporated in patients with *Klebsiella* sepsis [2]. Notably, this study reviewed 39 cases of EE and outcomes over a 10-year period. Of these cases, 45% were associated with *Klebsiella* infection; 95% of the *Klebsiella* patients possessed liver abscess, 60.0% required evisceration, and 20.0% had extreme vision loss to the point of inability to perceive any light. Therefore, given the high rate of vision impairment and visual morbidity in *Klebsiella* sepsis patients, implementation of eye screening such as a fundoscopic exam by an ophthalmologist even prior to the onset of visual symptoms may prevent irreversible vision loss in future patients.

Finally, it is interesting to note that the HvKP is a significant evolving subtype globally that has more severe complications (central nervous system disease, endophthalmitis) than the classic *Klebsiella pneumoniae* subtype [10]. The string test is a laboratory technique used to differentiate this hypervirulent strain, which demonstrates hyperviscosity with a hypermucoviscous appearance [11]. Our patient had a positive string test performed in the lab. This underscores the importance of this test as a viable option to differentiate between classic and hypervirulent strains of *Klebsiella*, which can guide early management to help limit serious complications.

## Conclusions

EE is a rare but serious complication of *Klebsiella* bacteremia with devastating irreversible long-term ocular complications if not promptly treated. Patients with clinical suspicion of endophthalmitis should be immediately started on antibiotics with urgent ophthalmology and infectious disease consultation. Vitreous fluid cultures are helpful but may not be a sensitive or specific test to rule out or rule in infection. Consideration should be given to performing an eye exam to rule out endophthalmitis in patients with *Klebsiella* bacteremia.
